# Associations between Social Support and Condom Use among Commercial Sex Workers in China: A Cross-Sectional Study

**DOI:** 10.1371/journal.pone.0113794

**Published:** 2014-12-01

**Authors:** Ren Chen, Feng Tao, Ying Ma, Liqin Zhong, Xia Qin, Zhi Hu

**Affiliations:** 1 Department of Epidemiology and Health Statistics, School of Public Health, Anhui Medical University, Hefei, Anhui, China; 2 School of Health Service Management, Anhui Medical University, Hefei, Anhui, China; University of Washington, United States of America

## Abstract

**Objective:**

The aim of this study was to investigate the association between social support and AIDS high-risk behaviors in commercial sex workers (CSWs) in China.

**Methods:**

A cross-sectional study was performed based on a convenience sample. Data were collected through questionnaire interviews including information about social demographic characteristics, the Social Support Rating Scale (SSRS) and AIDS knowledge. Multiple logistic regression was performed to evaluate the association between social support and AIDS high-risk behaviors, specifically condom use during commercial sex.

**Results:**

A total of 581 commercial sex workers from 4 counties in East China participated in the study. The majority of the participants were 15 to 30 years old (79.7%). Sources of individual and family support were mainly provided by their parents (50.3%), relatives and friends (46.3%), spouses (18.4%), respectively. Univariate analysis revealed that marital status, hobbies, smoking habit, individual monthly income and family monthly income were all significantly correlated with current levels of social support being received (*P* = 0.04, *P* = 0.00, *P* = 0.01, *P* = 0.01, *P* = 0.01, respectively). Furthermore, Multiple logistic regression analysis indicated that after adjusting for confounding factors, high levels of social support were significantly correlated with increased condom use at the last sexual encounter (*P* = 0.02, *OR = *1.86, 95%*CI*: 1.10–3.16); and consistently in the past month with clients (*P = *0.03, *OR = *2.10, 95%*CI*: 1.09–4.04).

**Conclusion:**

CSWs with high levels of social support are more likely to use condoms during commercial sex. This suggests that increasing social support can potentially reduce AIDS-related high-risk behaviors and accordingly play an important role in AIDS prevention.

## Introduction

As a nationwide epidemic in China, AIDS infection rate through sexual contact has kept dramatically increasing in recent years [1]–[4]. It has reported by the Ministry of Health of China that the new infection rate due to heterosexual transmission has grown from 40.3% in 2008 to 47.1% in 2009. In some regions of China, the prevalence of HIV infection among commercial sex workers (CSWs) is higher than 10% [5]–[8], strongly indicating that CSWs are a high-risk group and a potential “bridge” for HIV transmission [9]. A similar pattern is extensively observed worldwide [10]–[11] and CSWs have been the focus of AIDS prevention and control strategies. Heterosexual contact has been generally acknowledged as a major AIDS-related high-risk behavior and the use of condoms for penetrative sex is a key method to prevent HIV transmission [12]–[13].

One important aspect of social capital, social support, is defined as “the general or specific supportive behavior from people in the social network”, which can improve individuals' social adaptability, and the ability to cope with an adverse environment [14]. Typically these supportive behaviors are emotional, tangible and informative forms of support [15]. Social support is recognized as an important factor for myriad of health outcomes [16], e.g., cancer, heart disease, fracture, and rheumatoid arthritis. However, most studies have focused on social support with self-reported health, psychological and mental health [17]–[24], with a limited number of studies focusing on the role of social support and HIV/AIDS risk reducing behaviors [25]–[27]. The impact of social support on AIDS high-risk behaviors in commercial sex workers has rarely been reported in China. It is of great significance to explore the association of social support and AIDS-related high-risk behaviors in CSWs. In this study, we evaluated the hidden impact of social support on AIDS-related high-risk behaviors, specifically condom use among the CSWs.

## Methods

### Ethical Considerations and Informed Consent

Ethical approval for the study was obtained from the Biomedical Ethics Committee, Anhui Medical University. Respondents were verbally informed of the study purpose and procedures, sensitive questions, confidentiality, compensation, and their rights to refuse or quit the interview. Study participants expressed a verbal understanding of these issues and provided written consent. Eight respondents (1.9%) under the age of 18 participated in our study. Underage participants were required to provide personal written informed consent, as well as consent from their entertainment supervisors. Supervisors were allowed to provide consent on behalf of parents or guardians to protect the sex workers' identity and privacy. The owners or supervisors of the entertainment establishments were plausibly the caretakers or guardians of the minors. We explained the above situation to the Biomedical Ethics Committee of Anhui Medical University and obtained its approval.

### Participants, Study Design and Procedures

The participants in this study were female commercial sex workers in the adult entertainment industry. A cross-sectional convenience sampling strategy was carried out in four counties (Fuyang, Ma'anshan, Bengbu and Wuhu) in Anhui Province, China, in November 2010. Based on the geographic distribution of commercial sex workers in Anhui province, we selected one representative city from each of four geographic areas: Fuyang city in western Anhui; Ma'anshan city in eastern Anhui; Bengbu city in northern Anhui; and Wuhu city in southern Anhui. Staff from local Centers for Disease Control and Prevention contacted the owners or supervisors of the adult entertainment establishments to set up interviews with CSWs. CSWs were voluntarily asked to participate in anonymous surveys. Social demographics, social support being received, knowledge about AIDS, condom use, and other questions (e.g. reasons for not using condoms) were collected from the survey. Of the 56 total entertainment establishments surveyed, there were three sauna rooms, 11 bath centers, 21 outdoor bathing facilities, three KTVs (a for-profit music entertainment establishments and popular party spots in China), six nightclubs, four massage parlors, seven dance clubs, and one hotel; representing a comprehensive sample of the local adult entertainment industry. Among 594 participants interviewed, 581 questionnaires were valid with the response rate of 98%. One-to-one interviews with each CSW were conducted in a private room, lasting 0.5–1 hour. The surveys were anonymous. Personal status, social support being received, knowledge about AIDS, condom use, and a few sensitive questions (e.g. reasons for not using condom) were collected from the survey.

### Data Collection

Three questionnaires were used in this survey: a social demographics questionnaire, the Social Support Rating Scale (SSRS), and the AIDS KAP (knowledge, attitude and practice) questionnaires were introduced in this survey. Basic social demographic information consisted of age, marriage, education, hobby and income. Social support refers to assistance and protection provided to individuals. We measured social support using the Social Support Rating Scale (SSRS) [28]–[29], which has been used and verified among different Chinese populations [17]. The SSRS questions included: 1. *“How many close friends or relatives do you have?”* 2. *“What was your living situation in the past year?”* 3. *“How do you feel about your relationships with neighbors or friends?”* 4. *“How do you feel about your relationships with coworkers?”* 5. *“Did you receive any support and care from family members?”* 6. *“Who was the source of financial or material support when you were in need?”* 7. *“Who was the source of comfort and care when you were in trouble?”* 8. *“Did you have anyone to talk to when you were under stress?”* 9. *“How did you acquire help when you were in trouble?”* 10. *“How many times did you participate in group activities in the past year?”* The SSRS was scored as the following: (1) Questions 1–4 and 8–10 had single choice, while options 1, 2, 3, 4 corresponded to 1, 2, 3, 4 points respectively. (2) Question 5 had options A, B, C, or D, which were used to calculate the total score, with each response representing no support to full support (1–4 points, respectively). (3) Questions 6 & 7: no points were assigned to the answer “no source”, otherwise the number of points reflect the number of individuals providing support. The total score in the survey was 40 points, and a higher score indicated more social support. Scores were further divided into two groups, representing low social support (0 =  mean score of 0–20 points) and high social support (1 =  mean score of 21–40 points). AIDS KAP questionnaires included HIV-related high-risk behaviors [27], [30]. Respondents self-reported condom use at the last sexual encounter (yes  = 1, no  = 0) and the frequency of condom use in the past month (3 =  always, 2 =  often, 1 =  occasionally, 0 =  never).

### Data Analysis

All data were manually input into Epidata 3.0 software and were crosschecked and verified by trained staff. For descriptive statistics, the means and standard deviations (SD) were calculated using SPSS17.0 (SPSS Inc, Chicago, IL). To test the associations, univariable and multiple logistic regression were performed to assess the impact of social support on condom use. Statistical significance was determined by a P-value ≤0.05 (two-tailed).

## Results

### Social demographic characteristics of CSWs

Most of the participants mainly worked in nightclubs, bath centers and bars. 79.7% of the CSWs were at the age from 15 to 30, and 96.4% were of the Han ethnicity. 32.7% of CSWs were from other provinces (outside Anhui), suggesting high mobility of the CSWs. Among the CSWs surveyed, 60.1% were unmarried, 67.0% were below the education level of junior high school, 66.8% worked in commercial sex industry for less than a year, and 21.5% had religious belief. Most CSWs (97.9%) had hobbies, such as reading, watching TV, playing cards, and etc. The percentages of smoking and alcohol drinking were 52.0% and 42.3%, respectively. Both individual monthly income and family monthly income per person were mostly below 1000 Renminbi (15.7%, 20.0%, respectively). 45.0% were unemployed before entering into the sex industry. The results were summarized in Table 1.

**Table 1:** Social demographic characteristics of CSWs and univariable associations between social support and other co-variables.

		social support			
Variables	Variable categories	Low [n(%)]	High [n(%)]	*OR*	95%*CI*
Age(years)	15–30	50(10.8)	413(89.2)	1.00	Reference
	>31	20(16.9)	98(83.1)	0.59	0.34–1.04
Nationality	Han	66(11.8)	494(88.2)	1.00	Reference
	Minority	4(19.0)	17(81.0)	0.57	0.18–1.74
Province	Anhui Province	48(12.3)	343(87.7)	1.00	Reference
	Other Provinces	22(11.6)	168(88.4)	1.07	0.62–1.83
Marital status	Unmarried	34(9.7)	315(90.3)	1.00	Reference
	Married/divorced/widowed	36(15.5)	196(84.5)	0.59*	0.36–0.97
Education	Illiteracy/primary school	54(13.9)	335(86.1)	1.00	Reference
	Junior/senior high school/university	16(8.3)	176(91.7)	1.77	0.99–3.19
Time employed as a CSW (<1 year)	Yes	16(8.3)	177(91.7)	1.00	Reference
	No	54(13.9)	334(86.1)	0.56	0.31–1.01
Hobbies	No	5(41.7)	7(58.3)	1.00	Reference
	Yes	65(11.4)	504(88.6)	5.54**	1.71–17.96
Individual monthly income (Renminbi)	<1000	19(20.9)	72(79.1)	1.00	Reference
	≥1000	51(10.4)	439(89.6)	2.27*	1.27–4.07
Family monthly income per person (Renminbi)	<1000	26(22.4)	90(77.6)	1.00	Reference
	≥1000	44(9.5)	421(90.5)	2.06**	1.12–3.54
Religion	No	400(78.3)	56(80.0)	1.00	Reference
	Yes	111(21.7)	14(20.0)	1.05	0.53–2.05
Smoking	No	23(8.2)	256(91.8)	1.00	Reference
	Yes	47(15.6)	255(84.4)	2.05**	1.21–3.48
Drinking	No	38(11.3)	297(88.7)	1.00	Reference
	Yes	32(13.0)	214(87.0)	0.86	0.52–1.41
Previous occupation	Farmer/jobless	31(11.9)	230(88.1)	1.00	Reference
	Businessman/other	39(12.2)	281(87.8)	1.37	0.83–2.26

*P<0.05, **P<0.01.

### Social support and factor analysis

Among the 581 eligible participants, 55.6% kept in contact with three or more family members. 52.2% of the participants lived with their families or friends, and 68.7% felt greatly being cared for by their neighbors or friends. To avoid discrimination towards their occupation, 16.2% of the participants did not participate in any community activities in the past year. When facing hardships, 58.3% of the participants talked with only 1–2 close people, 15.2% proactively sought for understanding and support, but 26.5% never did. In addition, most of the participants concealed the fact that they were sex workers to their families. When having difficulties, 17.0% could not receive any care or financial support from their network to resolve issues The sources of individual and family help were mainly from their parents or other family members (50.3%), relatives and friends (46.3%), and spouses (18.4%).

Univariable logistic regression showed that compared with their respective control groups, social support was influenced by several factors, including marital status, education, hobbies, smoking and family income (Table 2). It was discovered that, as compared to the unmarried group, the married/divorced/widowed group were less likely to report social support (P = 0.04, OR = 0.59, 95%CI: 0.36–0.97). Participants with hobbies tended to have a higher level of social support than those without hobbies (P = 0.00, OR = 5.54, 95%CI: 1.71–17.96). Those who smoked had more social support (P = 0.01, OR = 2.05, 95%CI: 1.21–3.48). In terms of individual monthly income and family monthly income per person, the group with an income ≥1000 Renminbi obtained more social support (P = 0.01, OR = 2.27, 95%CI: 1.27–4.07; P = 0.01, OR = 2.06, 95%CI: 1.12–3.54, respectively).

**Table 2:** Association between variables and condom use by Binary logistic regression.

		Condom use at the last sexual encounter with clients			Consistent condom use in the past month with clients		
Variables	Variable categories	*P*	*OR*	95%*CI*	*P*	*OR*	95%*CI*
Age(years)	15–30	0.05	1.00	Reference	0.14	1.00	Reference
	>31		1.54	0.99–2.37		1.63	0.85–3.11
Nationality	Han	0.91	1.00	Reference	0.98	1.00	Reference
	Minority		1.05	0.43–2.58		1.02	0.29–3.52
Province	Anhui Province	0.06	1.00	Reference	0.90	1.00	Reference
	Other Province		1.42	0.99–2.04		1.03	0.63–1.69
Marital status	Unmarried	0.00**	1.00	Reference	0.01*	1.00	Reference
	Married/divorced/widowed		1.69	1.19–2.39		2.06	1.23–3.46
Education	Illiteracy/primary school	0.08	1.00	Reference	0.42	1.00	Reference
	Junior/senior high school/university		0.73	0.52–1.04		0.82	0.51–1.33
Time employed as a CSW(<1 year)	Yes	0.26	1.00	Reference	0.63	1.00	Reference
	No		1.22	0.86–1.74		0.89	0.54–1.46
Hobbies	No	0.44	1.00	Reference	0.30	1.00	Reference
	Yes		1.56	0.49–4.91		2.01	0.53–7.58
Individual monthly income (Renminbi)	<1000	0.32	1.00	Reference	0.21	1.00	Reference
	≥1000		1.26	0.80–1.98		1.45	0.81–2.6
Family monthly income per person (Renminbi)	<1000	0.34	1.00	Reference	0.21	1.00	Reference
	≥1000		0.86	0.63–1.17		0.78	0.53–1.15
Religion	No	0.93	1.00	Reference	0.64	1.00	Reference
	Yes		1.02	0.66–1.57		0.87	0.49–1.55
Smoking	No	0.00**	1.00	Reference	0.12	1.00	Reference
	Yes		1.62	1.16–2.26		1.45	0.91–2.31
Drinking	No	0.00**	1.00	Reference	0.00**	1.00	Reference
	Yes		2.82	1.97–4.03		2.88	1.68–4.94
Previous occupation	Farmer/jobless	0.37	1.00	Reference	0.20	1.00	Reference
	Businessman/other		1.16	0.83–1.63		1.35	0.85–2.15

*P<0.05, **P<0.01.

### Social support and condom use in CSWs

In this study, condom use was asked among CSWs. Condom use during the last sexual intercourse with a client was 60.78%, with 85.54% reporting consistent condom use in the past month. Client unwillingness to use a condom (38.73%), use of other contraception (15.80%) and no access to condoms (9.11%) were the reported reasons for having sex without a condom. Univariable binary logistic regression (Table 2) showed that marital status, smoking and drinking were associated with condom use during the last sexual intercourse. Marital status and drinking were associated with consistent condom use in the past month with clients (Table 2).

The multiple logistic regression implied that social support (divided into high vs. low social support groups) was significantly related to condom use during the last sexual intercourse and consistent condom use in the past month (Table 3 and 4). Condom use in the last sexual intercourse with a clients in the high social support group was 1.86 times higher than in the low social support group. Consistent condom use in the past month with clients in the high social support group was 2.10 times higher than in the low social support group. These results indicate that social support was positively correlated with condom use and was predictive of condom-protected sex.

**Table 3:** Associations between social support and condom use at the last sexual encounter with clients using multiple logistic regression.

	Condom use at the last sexual encounter with clients				Model1*	
Item	Yes [n(%)]	No [n(%)]	*P*	*OR*(95%*CI*)	*P*	a*OR*(95%*CI*)
High social support (n = 511)	318(62.2)	193(37.8)	0.05	1.65(0.99–2.72)	0.02	1.86(1.10–3.16)
Low social support (n = 70)	35(50.0)	35(50.0)		1.00 (Reference)		1.00(Reference)

*Adjusted for age, province, marital status, smoking and drinking.

**Table 4:** Association between social support and consistent condom use in the past month with clients using multiple logistic regression.

	Consistent condom use in the past month with clients				Model1*	
Item	Yes [n(%)]	No [n(%)]	*P*	*OR* (95%*CI*)	*P*	a*OR* (95%*CI*)
High social support (n = 511)	442(86.5)	69(13.5)	0.04	2.55(1.01–6.46)	0.03	2.10(1.09–4.04)
Low social support (n = 70)	55(78.6)	15(21.4)		1.00(Reference)		1.00(Reference)

*Adjusted for marital status and drinking.

## Discussion

Our study showed that, although most of the CSWs received relatively reasonably high social support, a considerable portion lacked adequate social support. The reasons cited for insufficient social support were manifold. Some CSWs may have been reluctant to seek social support due to prevailing discrimination in society towards sex workers and their avoidant personality [31]. More importantly, social support resources may have been limited or inaccessible. The mobility of CSWs may also limited their access to social support, especially support from their family members in their hometown [32]. In this study, many CSWs lived alone, far away from their families or had no families or relatives to contact for social support although family members were their main sources for social support. Family support has been reported s a key component to exuding a positive attitude towards life, which may play an important role to avert high-risk behaviors [33]–[34].

Several factors influenced the levels of social support received by CSWs. In terms of marital status, contrary to our hypothesis, unmarried CSWs reported higher social support than the married, divorced and widowed groups. One potential explanation could be that the unmarried CSWs had a larger social networks than the married ones. Another possibility is that the unmarried group suffered less violence from their stable partners. Because of their illegal or stigmatized status, about 58% of CSWs reported ever experiencing violence and psychosocial distress from their stable partners (e.g., husbands) [35]. Education was positively correlated with AIDS knowledge [36], and therefore should have increased the level of social support received. Surprisingly, education had no statistically significant role on the level of social support. It is possible that CSWs with a higher education level more likely depend on themselves, instead of seeking support from others. Compared to the control group, CSWs with no hobbies had less social support. Hobbies provide a conduit for social interaction and can increase communication with friends and coworkers and are used to acquire useful information. Hobbies and socialization could serve as outlets for CSWs to share their feelings, thoughts, and experiences regarding condom use with fellow CSWs. CSWs with individual monthly income ≥1000 Renminbi and family monthly income per person ≥1000 Renminbi were in the high social support group compared with <1000 group. This suggested that social support was proportionate to income and those who were in the most desperate financial situation were most willing to seek help.

The findings showed that both condom use during the last sexual encounter and consistent condom use in the past month with clients were much lower than that of other Asian countries [37], far below the goal of 100% CUP (100% condom use program) [38]–[40]. The most important reason cited for condom disuse was client refusal. Unprotected sexual intercourse increased not only the risk of HIV infection for the CSWs themselves, but also the risk of their spouses, boyfriends and other clients. Client refusal to wear condoms may have increased the magnitude of the HIV epidemic in China. It is critical to educate sex clients on the importance of condom use and safe sex practices. Furthermore, it would also be beneficial to improve the CSWs' negotiation skills for condom use with their clients through self-help groups [41]. In addition, we found that CSWs' condom use rate with clients was higher than that with stable sexual partners (spouses or boyfriends), both during the last sexual encounter and in the past month. Previous studies have found that CSWs were less likely to use condoms during sex with stable sexual partners than with their clients [42]–[43]. This phenomenon highlights the “bridging” role of commercial sex workers in the transmission of the HIV/AIDS.

We further explored the impact of social support on condom use in the CSWs. Our findings showed that social support was significantly related to condom use during the last sexual encounter and in the past month. Social support has been shown to be essential for the physical and mental health of CSWs [44], and is positively correlated with condom use. CSWs with high social support were more likely to communicate with friends and coworkers, participate in social activities, and seek care and encouragements [45]–[46]. Our results are consistent with a few previous findings that advocate for integrating social support into AIDS prevention [47]–[50]. It makes full sense that promotion of condom use by increasing maximal social support to CSWs could be one of the fundamental solutions to reduce the risk of HIV transmission [38], [51]; it thus is necessary to use this mechanism to develop HIV/AIDS prevention and control strategies in China.

This study contributes to the literature in a number of ways. Little literature exists on the social aspects of the AIDS epidemic. Our results demonstrate the associations between social support and safer sex. Given the ongoing HIV epidemic among female sex workers in China, this study provides evidence for using social support approach to reduce HIV infections among CSWs. However, more research needs to be done, as there are critical needs to verify the association between social support and condom use in large-scale studies, to estimate the size of female sex workers, and to identify their service location in order to design successful HIV/AIDS prevention programs. The government should establish and improve the social support system and provide alternative job opportunities and advancement life for CSWs [52], and periodically carry out health education services towards the prevention of HIV/AIDS [53]. At the same time, HIV/AIDS intervention work should consider the role of peer education organization to promote condom use and negate risky sexual behavior.

## Conclusions

In our study, we found that commercial sex workers with higher levels of social support had greater chance of condom use during commercial sex with clients. This suggests that more social support can potentially reduce AIDS-related risk behaviors and play an effective role in AIDS prevention. More comprehensive efforts should promote social support among CSWs to prevent HIV infection.

## Limitations

Our study also has several limitations, first, our sample was non-random and convenience sampling bias may have been introduced. Second, it was cross-sectional study, making it impossible to fully evaluate the CSWs. Third, as it was conducted in one province, the generalizability is limited. Large-scale studies are needed to verify the findings.
